# Comorbidity of Symptoms of Alcohol and Cannabis Use Disorders among a Population-Based Sample of Simultaneous Users. Insight from a Network Perspective

**DOI:** 10.3390/ijerph15122893

**Published:** 2018-12-17

**Authors:** Stéphanie Baggio, Marlène Sapin, Yasser Khazaal, Joseph Studer, Hans Wolff, Gerhard Gmel

**Affiliations:** 1 Division of Prison Health, Geneva University Hospitals and University of Geneva, 1226 Thônex, Switzerland; hans.wolff@hcuge.ch; 2 Life Course and Social Inequality Research Centre, University of Lausanne, 1015 Lausanne, Switzerland; 3 Swiss Center of Expertise in Social Sciences (FORS) & Swiss National Centre of Competence in Research “LIVES—Overcoming Vulnerability: Life Course Perspectives”, University of Lausanne, 1015 Lausanne, Switzerland; marlene.sapin@unil.ch; 4 Geneva University Hospitals, 1205 Geneva, Switzerland; yasser.khazaal@hcuge.ch; 5 Alcohol Treatment Centre, Lausanne University Hospital CHUV, 1011 Lausanne, Switzerland; joseph.studer@chuv.ch (J.S.); gerhard.gmel@chuv.ch (G.G.); 6 Addiction Switzerland, 1001 Lausanne, Switzerland; 7 Centre for Addiction and Mental Health, M6J 1H4 Toronto, Canada; 8 University of the West of England, BS16 1QY Bristol, UK

**Keywords:** addiction, alcohol, cannabis, marijuana, polydrug use

## Abstract

Research into comorbidity of alcohol and cannabis use disorders has resulted in inconsistent findings, especially among simultaneous users, who used alcohol and cannabis together on a single occasion. This study investigated the association of alcohol and cannabis use disorders among simultaneous users using a network perspective, which considers direct relationships between symptoms. We used a subset of simultaneous alcohol and cannabis users driven from the representative population-based sample of young Swiss men cohort study on substance use risk factors (C-SURF) (*n* = 1559 at baseline and *n* = 991 at follow-up). Self-reported symptoms of alcohol and cannabis use disorders were collected. Network analyses included network estimation, visualization, and community detection tests. Alcohol and cannabis use symptoms were separated in two distinct clusters, with few paths between them (eleven positive edges at baseline, three at follow-up). Withdrawal symptoms were likely to connect the two disorders at baseline, but not at follow-up. Alcohol and cannabis use disorders appeared as separate disorders among simultaneous users. Our findings mitigated previous findings on the detrimental association between alcohol and cannabis use. Future studies should incorporate network analyses as a means to study comorbidity in other community and clinical samples to confirm our preliminary findings.

## 1. Introduction

Alcohol and cannabis is a common polydrug combination, and the potential detrimental effects on health of both drugs are elevated when the two types of substances are co-ingested (i.e., used at the same time so the effects of the two substances overlap) [1,2]. This definition allows capturing the interacting effects of substances, because the effects of the substances overlap, with a substance that may increase or decrease the effect of the other. Such combinations should be taken into account when studying polysubstance use, to achieve a better understanding of their detrimental health associations. Compared with the use of alcohol and cannabis separately, the use of both substances on a single occasion is associated with higher levels of substance use, engagement in risky or norm-violating behaviors, and more substance-related problems, such as educational, legal, relational, and health problems [3,4,5,6,7]. Simultaneous alcohol and cannabis use is also likely to increase the severity of alcohol and cannabis use disorders [6,8]. Therefore, simultaneous use should be an important public health concern.

Moreover, research on the relationships between alcohol and cannabis use has yielded inconsistent findings [9,10,11]. There is a general acknowledgement on the fact that alcohol and cannabis use disorders are separate syndromes [12]. However, when individuals use both substances (concurrently or simultaneously), cannabis is sometimes described as a substance that complements alcohol use and, at other times, as a substitute for alcohol use, while other studies have supported neither or both of these conceptions [9]. These findings suggest that alcohol and cannabis use disorders may be interacting disorders. Research based on interventions’ outcome have also reported inconsistent findings. Some studies concluded that cannabis use, and even low cannabis intake, was associated with a lower percentage of days of abstinence [10,12], but others showed that cannabis use did not decrease the efficacy of alcohol interventions [13,14]. Despite the absence of convincing evidence on the relationship between alcohol and cannabis use, complete abstinence from both substances is commonly recommended in treatment [11]. Data-driven approaches of the comorbidity of alcohol and cannabis use are therefore needed to achieve a better understanding of their relationships and, ultimately, to provide guidance for treatment. Simultaneous users should be at special focus because this is a common pattern of substance use, and because disorders may be strongly interacting when both substances are used on the same occasion [2,8].

Investigating network structures to understand the relationships between symptoms of a disorder is a recent research field in clinical psychology and psychiatry [15,16]. The network perspective differs drastically from standard approaches, and has contributed to mental health research by tackling several research questions related to the associations between several symptoms of a unique disorder, but also in the case of comorbidity [17]. The network perspective supposes that a disorder is a dynamic system or network composed of symptoms that are directly related to one another [18]. Therefore, a disorder is no longer considered as a latent construct that cause symptoms: it is composed of the symptoms themselves and of their direct relationships. The approach supposes that symptoms cluster in a nonarbitrary way, with direct and potentially causal symptom–symptom relationships [19]. They are mutually interacting and may also be reciprocally reinforcing [19]. Therefore, it offers a new way to understand disorders [16]. Studying the comorbidity of two or more disorders from a network perspective entails two major advantages. First, it allows investigating the extent to which these two disorders have clear boundaries. If two disorders are clearly separated, one would expect two separate clusters of symptoms. Second, it is possible to explore pathways between the two disorders, with the potential existence of so-called bridge symptoms that create a path between two disorders [17,20]. For example, a previous study investigating the comorbidity of problematic internet use and problem gambling concluded that problem gambling and problematic internet use were separate disorders, but more strongly related for online gamblers in comparison with land-based gamblers [19]. To answer this research question, the authors created two networks of symptoms of problematic internet use and problem gambling, one for online gamblers and one for land-based gamblers, and tested the strength of the relationships between disorders. Such studies provide a better overview of the relationships of multiple disorders and how they may interact and overlap. Thus, the network perspective offers a straightforward way to examine how different disorders may co-occur in a network structure, with interactions occurring between symptoms, possibly irrespective of disorder boundaries [21,22]. Therefore, network-based analyses may provide new insights into the comorbidity of alcohol and cannabis use disorders. This perspective overcomes some issues of standard approaches, for example, considering that symptoms are passive indicators of the syndrome and are independent from each other. 

Few studies have applied network analysis to the study of substance use disorders. An exploratory study focused on separate networks for different substances and showed that some symptoms of substance use disorders were more important (central) than others [23]. This study provided a first glance into the structure of substance use disorders’ networks, but more investigations are needed to examine the relationships between several substance use disorders, especially among young adults for whom they represent a prevalent form of psychopathology [24,25]. Another study investigated threshold of alcohol use disorder symptoms, but did not focus on comorbidity with other substance use disorders [26].

The aim of this exploratory data-driven study was to use network analysis to investigate the comorbidity of alcohol and cannabis use disorders among simultaneous alcohol and cannabis users from a population-based sample of young men. Specifically, we investigated the extent to which (1) the symptoms of these two substances use disorders overlapped; and (2) whether some bridge symptoms between disorders could be identified.

## 2. Materials and Methods 

### 2.1. Sample and Procedures

Data were collected in the two first waves of the cohort study on substance use and risk factors (C-SURF) [27], a longitudinal study designed to assess substance use patterns and associated consequences among young Swiss men. Participants were enrolled during mandatory conscription in three Swiss national military recruitment centers. There was no preselection for this conscription, so all young men around 20 years old were eligible for study inclusion. Participation was independent from the military recruitment. The three recruitment centers cover 21 out of 26 cantons of the country, including French- and German-speaking participants. A previous study on non-response in C-SURF found the non-response bias to be small [28]. All subjects gave their informed consent for inclusion before they participated in the study. The study was conducted in accordance with the Declaration of Helsinki, and the protocol was approved by the Lausanne University Medical School’s Clinical Research Ethics Committee (No. 15/07).

Of the 7556 conscripts who gave written consent to participate, 5987 (79.2%) filled in the baseline questionnaire between September 2010 and March 2012. The follow-up took place, on average, 15 months later (2012–2013). A total of 6020 participants completed the follow-up questionnaire, including participants who did not answer the baseline questionnaire. More detailed information on the sample is available elsewhere [27]. As our secondary-data-analysis study focused on simultaneous alcohol and cannabis use, we selected participants who used both alcohol and cannabis in the same occasion. Participants were asked the following question in the baseline and follow-up questionnaire: “how often did you take alcohol along with cannabis (simultaneously) in the past 12 months? By “simultaneously”, we meant shortly before or after drinking alcohol (in the same evening), but not the day after nor the day before”. Responses were collected on a six-point scale (“almost always”, “often”, “more or less half of the time”, “seldom”, “hardly ever”, and “never”). Participants who answered “hardly ever” or more were considered as simultaneous users. Of the 1752 participants who used both alcohol and cannabis at baseline, 94.1% reported simultaneous use of alcohol and cannabis (*n* = 1649). The sample of concurrent alcohol and cannabis users was too small to perform network analyses. Missing values (*n* = 90) were listwise-deleted, leading to our final sample of 1559 participants, which represents 95% of the simultaneous alcohol and cannabis users at baseline. At follow-up, among the 1577 alcohol and cannabis users, 94.9% reported simultaneous use (*n* = 1496). We deleted 24 missing values (*n* = 1472). The follow-up analysis included participants who were simultaneous users at both baseline and follow-up (*n* = 991, 67.3% of the follow-up sample) to see how alcohol and cannabis symptoms were associated for consistent simultaneous users over time.

### 2.2. Measures

*Alcohol use disorder symptoms*. Participants filled out questions related to the eleven criteria of the alcohol use disorder as reported in the fifth version of the diagnostic and statistical manual of mental disorders (DSM-5) (American Psychiatric Association, [29]) for the 12 previous months (see symptoms in Table 1). Symptoms were collected on dichotomous scales, in terms of presence versus absence and collected at baseline and follow-up. The reliability of the scale was acceptable: Kuder–Richardson Formula 20 = 0.73 and Spearman–Brown split-half correlation = 0.76.

*Cannabis use disorder symptoms*. Respondents answered the 10 criteria of the cannabis use disorder identification test-revised (CUDIT) [30] for the 12 previous months. For each of the 10 items, we recoded the responses into dichotomous symptoms: “never” = “absence”; “less than a month” or more = “presence”; for questions one and two, the lowest category “less than a month” and “one or two h” were considered as “absence”, and the other categories as “presence” (no use was not possible as people who complete the scale are cannabis users) instead of keeping the original four-point scale, in order to consider equivalent dichotomous low-threshold measures for alcohol and cannabis [26]. Symptoms were also collected at baseline and follow-up. The reliability of the scale was also acceptable: Kuder–Richardson Formula 20 = 0.74 and Spearman–Brown split-half correlation = 0.82.

Alcohol and cannabis use disorders were assessed in French or German. The psychometric properties were acceptable for both disorders and both languages. We performed confirmatory factor analyses for ordinal data (weighted least squares means and variances, WLSMV estimation) to confirm the single factor for alcohol and cannabis use disorders. Analyses were conducted separately for French and German languages using Mplus 7 (Muthén & Muthén, Los Angeles, CA, USA). Fit indices were acceptable: root mean square error of approximation (RMSEA) ranged between 0.030 and 0.082, comparative fit index (CFI) between 0.969 and 0.984, and weighted root mean square residual (WRMR) between 0.858 and 1.519.

### 2.3. Data Analysis

After computing descriptive statistics for alcohol and cannabis use disorder symptoms, we estimated the symptoms’ network structure with the IsingFit method in R [31]. This method is designed for binary variables. It computes pairwise conditional associations between nodes, with a penalty weight to shrink the smalls coefficients to zero [32]. The network estimation combines L1-regularized logistic regression with model selection using the extended Bayesian information criterion [31]. To transform raw data into an adjacency matrix, two parameters are computed: an interaction parameter (which provides the strength of the interaction between two variables) and the node parameter (which gives the autonomous preference for each variable to take the value 1). These parameters are computed using iterative logistic regressions, each variable being regressed on all others, with a L1-penalty imposed on each regression coefficient. Variables are defined as nodes (vertices in the adjacency matrix) and relevant relationships between nodes as edges (undirected graph).

To investigate whether symptoms of alcohol and cannabis use disorders were separate from one another, and we applied community detection analysis to identify the clusters of symptoms in the global network of alcohol and cannabis symptoms, based on the walktrap community finding algorithm. This algorithm identifies densely connected subgraphs using short random walks [33].

Finally, to identify potential bridge symptoms between alcohol and cannabis use disorders, we computed bridge centrality indices [34]. They indicate whether some symptoms have notable relationships with the other cluster of symptoms. Bridge strength is defined as the sum of the absolute weights of a focal symptom with all symptoms that are not in the same cluster. Bridge betweenness is the number of shortest paths going through a focal symptom that connect pairs of symptoms from different clusters. Bridge closeness is the inverse of the sum of shortest distances from a focal symptom to all other symptoms in the other cluster. Symptoms with high scores on bridge centrality indices indicate potential bridge symptoms, connecting symptoms of alcohol and cannabis use disorders. A higher score indicates a more important bridge centrality.

We checked for model accuracy using the recommended analysis [35]: edge weight accuracy. The edge weight accuracy was tested by drawing bootstrapped 95% confidence intervals. Overlapping confidence intervals between the different edges of the network mean that even if some edges may seem stronger, they are actually not significantly different. It does not affect the interpretation of the edges’ presence: an edge between two nodes means that the corresponding symptoms are connected. No test of model accuracy is available for bridge centrality indices, so these results should be interpreted carefully.

All analyses were performed twice: first, a baseline network, including symptoms of alcohol and cannabis at baseline for baseline simultaneous use; and second, a follow-up network, including symptoms at follow-up. In the second analysis, we used the subsample of participants who were simultaneous users of alcohol and cannabis at baseline and follow-up (*n* = 991). 

As a sensitivity analysis, we tested networks using the same symptoms for the two disorders (and excluding items that were not assessed for both disorders), namely: excessive use, continued use, loss of control, loss of interest, withdrawal, health consequences, and social consequences (alcohol tolerance, craving for alcohol, frequency of cannabis use, and cannabis mood modification were not included). The results were very similar to those presented below, so we kept the whole scales for both disorders. Data are available upon request to the corresponding author. We also tested other cut-off scores to dichotomize the CUDIT scale, with similar results.

We used R 3.3.2 for all analyses, with the package IsingFit 0.3.1 and qgraph 1.4.2 to visualize networks [36], the algorithm walktrap.community from the igraph 1.0.1 package to detect community, the package networktools 1.1.1 to compute bridge centrality indices, and the package bootnet 0.4 for bootstrap estimations [35].

## 3. Results

Participants were on average 20.0 ± 1.2 years old at baseline (21.3 ± 1.2 at follow-up); 57.6% were French-speaking, and 42.4% were German-speaking. Percentages of symptom endorsement are reported in Table 1. 

### 3.1. Baseline Network

**Figure 1:**
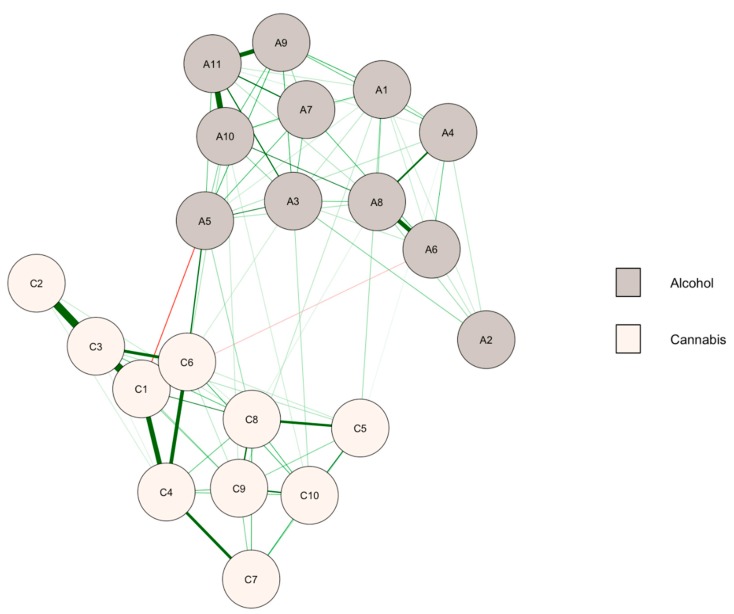
Network of alcohol and cannabis symptoms at baseline (*n* = 1559). A1–A11: symptoms of alcohol use disorder, C1–C10: symptoms of cannabis use disorder (see Table 1 for labels). Green (or red) paths are positive (or negative) regularized logistic regression weights. Thicker edges indicate a stronger relationship between symptoms. Node colors are defined according to the community detection analysis.

The association between cannabis and alcohol symptoms at baseline is presented in Figure 1, where we can see that few relationships linked alcohol and cannabis symptoms. The community detection analysis confirmed that the network was separated in two clusters, corresponding to the symptoms of alcohol use for the first one, and cannabis use for the second. There were 11 positive edges, of the 110 possible ones, between the symptoms of the two disorders. Therefore, only 10.0% of the possible edges connected the symptoms of two disorders. There were also two negative edges. The edge weight accuracy suggested that the strength of the edges should be compared cautiously because of overlapping confidence intervals, but within-cluster edges tended to be significantly higher than between-cluster edges (data available on request). 

Results on the centrality bridge indices of the symptoms (see Table 1) indicated that withdrawal connected the clusters of alcohol and cannabis symptoms (A5 and C6). These symptoms were potential bridge symptoms between the two clusters.

### 3.2. Follow-Up Network

**Figure 2:**
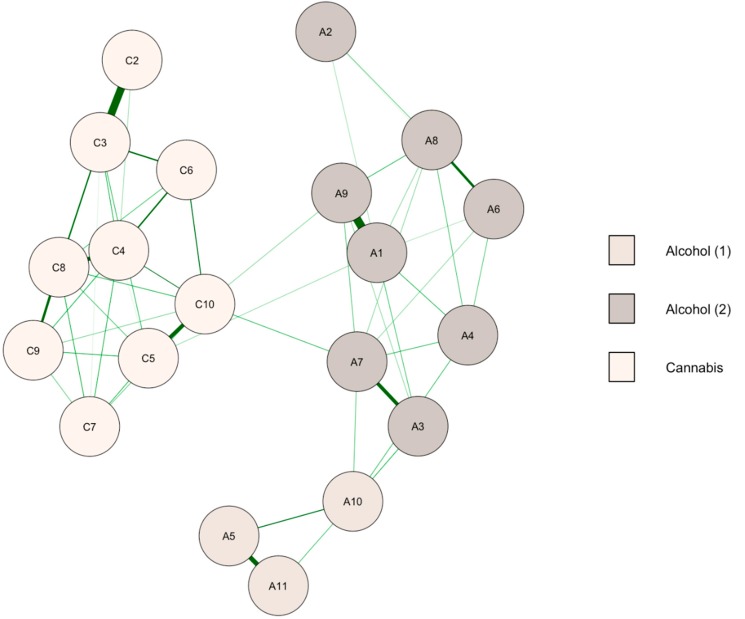
Network of alcohol and cannabis symptoms at follow-up (*n* = 991). A1–A11: symptoms of alcohol use disorder, C1–C10: symptoms of cannabis use disorder (see Table 1 for labels). Green paths are positive regularized logistic regression weights. Thicker edges indicate a stronger relationship between symptoms. Node colors are defined according to the community detection analysis.

The network of alcohol and cannabis symptoms at follow-up for participants who were simultaneous users at both baseline and follow-up is reported in Figure 2. The community detection analysis identified three clusters. Alcohol and cannabis symptoms were again separate clusters, and there was, in addition, two distinct clusters for alcohol use disorder. There were only three positive edges between alcohol and cannabis, i.e., 2.7% of all possible relationships between the two disorders. The edge weight accuracy suggested that the strength of the edges should be compared cautiously because of overlapping confidence intervals. 

Centrality bridge indices are reported in Table 1 (bridge indices were computed between alcohol use disorder including its two clusters and the cluster of cannabis use disorder). Withdrawal was no longer a potential bridge symptom between the disorders. Symptoms with the highest bridge indices were trying to cut down alcohol (A7) and difficulties in school/work related to cannabis use (C10).

## 4. Discussion

By investigating symptoms irrespectively of their disorders’ boundaries [21,22], this research provided a new vision of the comorbidity between symptoms of alcohol and cannabis use disorders among young men from a population-based sample who used them simultaneously. 

Our exploratory study showed that simultaneous alcohol and cannabis use was a frequent pattern of substance use in young adulthood, which was not associated with a common syndrome of substance use disorder. Indeed, the network analyses highlighted that symptoms of cannabis and alcohol use disorders were separate clusters with only a small number of paths connecting the two disorders. Overall, these results mitigated findings of recent studies suggesting that cannabis and alcohol use are interacting disorders [11]. If the disorders were interacting among simultaneous alcohol and cannabis users, we could expect strong between-disorders relationships, but it was not the case. Moreover, we could not highlight long-term associations between the disorders. Previous studies did not investigate direct relationships between the disorders and often focused on treatment outcomes such as abstinence and substance use-related problems. On the contrary, when studying direct relationships between symptoms from a network perspective, alcohol and cannabis use disorders did not appear as a unitary syndrome [12]. 

The most important relationship between the disorders at baseline was related to withdrawal symptoms. We identified alcohol and cannabis withdrawal as possible paths between disorders. When simultaneous users refrain from using alcohol and cannabis at the same time, they may experience several withdrawal symptoms (e.g., depressed mood, irritability, anxiety, sleep difficulty) that are not specific to alcohol or cannabis. Therefore, it is possible that disentitling alcohol- and cannabis-related withdrawal was not possible, resulting in a strong relationship between these two symptoms at baseline. Another explanation might be that individuals who experience alcohol withdrawal use cannabis to cope with withdrawal symptoms (or the opposite). However, this relationship was no longer highlighted at follow-up. As the replicability of centrality indices has been criticized, further studies should focus on withdrawal symptoms to test again our baseline findings [37,38,39]. This limitation does not affect the network structure (i.e., presence of edges and clusters), because the global characteristics of the network models have been described as consistent across methods and samples [37]. In addition, our follow-up analysis showed that our findings on the separate clusters for alcohol and cannabis use disorders seemed robust. 

One important difference between previous studies and our study was that we did not focus on a clinical sample, but on a population-based sample of young Swiss men. Clinical samples are often biased, since only a small proportion of addicted users seek help and treatment. Using large samples from the general population to assess substance use disorders is probably a reliable way to reach all sorts of substance users and to get a general picture of the relationships between alcohol and cannabis use. Our results are in line with previous research based on population-based samples showing that cannabis users respond similarly to alcohol interventions as non-cannabis users [40]. This also suggested that alcohol and cannabis use are not strongly interacting conditions. 

Focusing on simultaneous users seems crucial for future researches in the addiction field, in both population-based and clinical samples. Clinical trials designed to evaluate substance abuse treatment often exclude multiple drug users, and focus on a single-drug use type [8,41]. Since multiple drug use is common and harmful, it should not been seen as a hindrance in clinical trials [42]. The insights of this research may also be of use from a clinical perspective, and to provide guidance for substance abuse treatment. Drug abuse treatment programs traditionally recommend complete abstinence because of a fear that users will switch to another substance [10]. Indeed, the absence of abstinence is often described as a barrier to treatment, leading to delays in treatment initiation and relapse [11]. Besides, it is likely to decrease the willingness to seek treatment among users of these substances [10]. Our results on the relative independence of the two conditions among simultaneous users may be useful to develop guidance for substance use treatment. 

In the current state of the art, studies using network-based analyses mainly focused on group-level networks [43]. An interesting contribution of the network perspective is to identify symptoms that may predict the development of the disorder(s), i.e., symptoms that are highly connected in the network. These symptoms may provide early warning signals usable at the individual level, and may thus have direct therapeutic implications [16]. The network perspective appears as a helpful complement to standard analyses to guide clinical decision-making and treatment [44].

This study had some shortcomings. A first limitation was that the study only included men in their earlies twenties and substance use behaviors are distinct for women [45]. Data among women and older adults are needed to confirm our findings. Meanwhile, we should consider our conclusions as preliminary ones. A second limitation was that the study used self-reported scales, which may cause response bias and misunderstanding of the symptoms, especially for alcohol [46]. Young people are likely to misinterpret survey questions and share a misperception of alcohol symptoms, such as aftereffects and acute intoxication. Therefore, they might overreport physiological symptoms of withdrawal and tolerance [47]. For example, tolerance is often overreported in self-reported data. In our network, overreporting symptoms might have led to an artificial increase of relationships between symptoms. Another important limitation was that we used different tools to assess alcohol and cannabis use disorders. Even if we addressed this issue using a sensitivity analysis selecting common symptoms, further studies should use the same criteria for both disorders. In addition, the CUDIT also includes questions that are not symptoms of cannabis use disorder (e.g., felt stoned after using cannabis for three hours or more) and some symptoms considered low use (lower than monthly use and felt stoned for one or two hours) as “absence”. However, the sensitivity analysis focusing on the same symptoms for both disorders yielded similar results. Therefore, we are confident that our findings can be interpreted as investigating relationships between symptoms of the two disorders. Further studies are needed to confirm our results, and should include women, use clinical interviews, as well as different populations, such as older adults or treatment-seeking populations [48]. Furthermore, comparisons between simultaneous and concurrent users would provide more evidence of influence of simultaneous use on the relationship between disorders, beyond our descriptive findings (are the relationships between disorders stronger for simultaneous users compared to concurrent users or not?).

## 5. Conclusions

In conclusion, this study showed that alcohol and cannabis use disorders were distinct clusters of symptoms in the network analysis, suggesting thereby that they were not interacting disorders. Overall, network-based analyses appeared to be a promising new research perspective in mental health research, which emphasize the relationships between and within mental health disorders.

## Figures and Tables

**Table 1:** Percentages and bridge centrality of alcohol and cannabis use disorder symptoms.

Label	Items	Baseline (*n* = 1559)				Follow-up (*n* = 991)			
		%	Bridge Strength	Bridge Betweenness	Bridge Closeness	%	Bridge Strength	Bridge Betweenness	Bridge Closeness
A1	Neglect important activities	20.9	0.60	8	0.23	19.5	0.33	5	0.24
A2	Increased chances of getting injured	45.4	0.00	0	0.18	46.6	0.00	0	0.13
A3	Resume drinking habits despites problems with others	8.1	0.50	11	0.28	6.5	0.00	0	0.27
A4	Tolerance	34.2	0.07	0	0.19	27.4	0.00	0	0.22
A5	Withdrawal	6.9	2.09	69	0.40	4.7	0.00	0	0.16
A6	Drink more/longer than intended	43.0	0.28	0	0.18	51.2	0.00	0	0.17
A7	Try to cut down but couldn’t	5.8	0.00	9	0.25	5.5	0.63	54	0.35
A8	Spend time obtaining, using, recovering from alcohol	26.6	0.12	2	0.19	27.0	0.00	4	0.18
A9	Give up activities	5.0	0.00	14	0.26	4.5	0.37	17	0.25
A10	Continue drinking despites health problems	5.4	0.72	3	0.22	3.8	0.00	18	0.20
A11	Strong desire or urge to drink	6.0	0.00	0	0.24	6.0	0.00	0	0.15
C1	Frequency of cannabis use previous 12 months	48.6	1.11	0	0.24	100 *	-	-	-
C2	Felt stoned after using cannabis ≥3 h	37.4	0.00	0	0.21	47.4	0.00	0	0.16
C3	Felt stoned for ≥6 h	46.3	0.00	11	0.24	52.3	0.00	11	0.18
C4	Being not able to stop using cannabis	20.3	0.00	13	0.24	18.9	0.00	0	0.22
C5	Failed to do what is expected	36.0	0.43	3	0.21	35.2	0.33	17	0.25
C6	Need of cannabis in the morning after a heavy cannabis intake	16.6	1.36	64	0.31	12.9	0.00	18	0.22
C7	Felt guilty or remorseful	29.3	0.00	0	0.20	27.6	0.00	0	0.18
C8	Had a problem with memory/concentration	39.3	0.51	2	0.22	36.8	0.00	0	0.21
C9	Refrained from leisure activities	18.4	0.47	0	0.20	18.9	0.00	0	0.18
C10	Had difficulties at work/school	12.4	0.50	4	0.23	8.8	1.00	78	0.31

A: alcohol, C: cannabis. * This symptom was not included in the network analysis because it has no variance (symptom endorsed by all participants).
